# Second generation laryngeal mask airway during laparoscopic living liver donor hepatectomy: a randomized controlled trial

**DOI:** 10.1038/s41598-021-83173-5

**Published:** 2021-02-11

**Authors:** Doyeon Kim, Sukhee Park, Jong Man Kim, Gyu Seong Choi, Gaab Soo Kim

**Affiliations:** 1 Department of Anesthesiology and Pain Medicine, Samsung Medical Center, Sungkyunkwan University School of Medicine, 81 Irwon-ro, Gangnam-gu, Seoul, 06351 Republic of Korea; 2 Department of Anesthesiology and Pain Medicine, International St. Mary’s Hospital, Catholic Kwandong University School of Medicine, Incheon, Republic of Korea; 3 Department of Surgery, Samsung Medical Center, Sungkyunkwan University School of Medicine, Seoul, Republic of Korea

**Keywords:** Health care, Medical research

## Abstract

The second-generation laryngeal mask airway (LMA) provides a higher sealing pressure than classical LMA and can insert the gastric drainage tube. We investigated the difference in respiratory variables according to the use of second-generation LMA and endotracheal tube (ETT) in laparoscopic living liver donor hepatectomy (LLDH). In this single-blind randomized controlled trial, intraoperative arterial carbon dioxide partial pressure at 2 h after the airway devices insertion (P_a_CO_2__2h) was compared as a primary outcome. Participants were randomly assigned to the following groups: Group LMA (n = 45, used Protector LMA), or Group ETT (n = 43, used cuffed ETT). Intraoperative hemodynamic and respiratory variables including mean blood pressure (MBP), heart rate (HR), and peak inspiratory pressure (PIP) were compared. Postoperative sore throat, hoarseness, postoperative nausea and vomiting (PONV), and pulmonary aspiration were recorded. The P_a_CO_2__2h were equally effective between two groups (mean difference: 0.99 mmHg, *P* = 0.003; 90% confidence limits: − 0.22, 2.19). The intraoperative change in MBP, HR, and PIP were differed over time between two groups (*P* < 0.001, *P* = 0.015, and *P* = 0.039, respectively). There were no differences of the incidence of postoperative complications at 24 h following LLDH (sore throat and hoarseness: *P* > 0.99, PONV: *P* > 0.99, and *P* = 0.65, respectively). No case showed pulmonary aspiration in both groups. Compared with endotracheal tube, second-generation LMA is equally efficient during LLDH. The second-generation LMA can be considered as the effective airway devices for securing airway in patients undergoing prolonged laparoscopic surgery.

**Trial Registration** This study was registered at the Clinical Trial Registry of Korea (https://cris.nih.go.kr. CRiS No. KCT0003711).

## Introduction

General anesthesia is essential for the upper abdominal surgery. Endotracheal tube (ETT) is widely used for airway maintenance. Since laryngeal mask airway (LMA) was developed in 1980s^1^, it is frequently used for airway maintenance.

LMA has several advantages over ETT including ease of insertion, lower risk of trauma to the trachea, reduced postoperative sore throat, dysphagia, and dysphonia, and improved hemodynamic and respiratory stability during device insertion and anesthetic emergence^2^. However, due to the structural limitation of LMA, it might not provide proper seal and there are possibility of inadequate ventilation, hypoxia, and pulmonary aspiration compared to ETT^3,4^. Thus, LMA use has been limited to the surgery of short duration. In addition, obesity, laparoscopic surgery, and gastroesophageal reflux disease (GERD) have been considered as relative contraindications of LMA^5^.

Previous study has shown that both classic LMA and second-generation LMA did not increase the risk of gastric distension compared to ETT in gynecologic laparoscopy performed within 42 min^6^. In our institute, second-generation LMA has been used during laparoscopic liver resection since 2017. Moreover, we reported a case of successful application of second-generation LMA during laparoscopic living donor right hepatectomy^7^. Although the second-generation LMA with modified design is known to provide higher sealing pressure and reduces the risk of pulmonary aspiration, there is a lack of prospective research investigating the suitability of LMA in prolonged laparoscopic surgery.

Donor safety is a major concern of living-donor liver transplantation. Anesthesia and related complications, as well as short- to long-term morbidity and mortality, are issues to be addressed in terms of donor safety. The aim of present study was to evaluate the suitability of second-generation LMA in donors underwent laparoscopic living liver donor hepatectomy (LLDH).

## Methods

### Study design and participants

This single center, single-blinded, randomized controlled equivalance trial was approved by the Institutional Ethics Committee (Samsung Seoul Hospital Institutional Review Board, Seoul, Republic of Korea; Approval number: IRB No. SMC 2018-03-014) and written informed consent was obtained from all participants. It registered at Clinical Research Information Service (https://cris.nih.go.kr/cris/ CRiS No. KCT0003711, date of registration: 03/04/2019, principal investigator: Gaab Soo Kim,). In addition, we followed to the declaration of Helsinki.

Living liver donors over 18 years who underwent elective LLDH were recruited from July 2018 to September 2019. Exclusion criteria were American Society of Anesthesiologists (ASA) physical status ≥ III, body mass index (BMI) > 30 kg/m^2^, those who taken histamine 2-receptor blocker or proton pump inhibitor due to GERD, those who had upper airway deformity, recent upper respiratory infection, major oro-laryngeal surgery, and mouth opening < 3 cm.

### Randomization and blinding

Participants were randomly assigned to one of two study groups according to the use of airway device: group LMA *vs* group ETT. Random allocation was performed under 1:1 ratio with six blocking combination (4 numbers per block) using computer-generated program and the results were concealed to participants until each participant’s enrollment. Randomization was also maintained until opening a sealed opaque envelope immediately before surgery by one anesthesiologist (GSK). GSK, has extensive experience for second-generation LMA, performed management from anesthetic induction to removal of airway device and was not involved in data collection. LLDH was performed by two surgeons (JMK, GSC) blinded to the airway device. Although the attending anesthesiologist could not keep masked due to the nature of our trial, participants were masked until the completion of the study.

### Procedures

In the operating room, standard monitors were applied including electrocardiogram, pulse oximetry, and non-invasive blood pressure. Before anesthetic induction, midazolam 2 mg was injected and morphine sulfate 400 mcg was intrathecally given for postoperative analgesia. After preoxygenation for 3 min. anesthesia was induced with thiopental 5 mg/kg, vecuronium 1 mg/kg, remifentanil 0.2 mcg/kg/min, and isoflurane 2 vol%. Second generation LMA (LMA Protector Airway, Teleflex Medical Europe Ltd., Westmeath, Ireland) or ETT (Shiley Cuffed Basic Endotracheal Tubes, Medtronics, Minneapolis, USA) was placed according to the assigned study groups. The size of LMA was chosen according to the manufacturer’s recommendation and the size of ETT was chosen based on the gender (female: 7.0 mm internal diameter, male: 8.0 mm internal diameter, respectively). In the group LMA, the head of participant was in a sniffing position and the anesthesiologist secured jaw thrust and mouth opening with the left hand. LMA was advanced through the palate-pharyngeal curve with fully deflated status. LMA cuff was inflated and maintained within green zone and oropharyngeal leak pressure (OLP) was measured. OLP was assessed at the equilibrium airway pressure measured after applying a closed valve system with 4 L/min of oxygen with gas leakage occurred into mouth^8^. In the group ETT, tracheal intubation was performed using conventional laryngoscopy. The cuff pressure was maintained at 25 cmH_2_O in group ETT. The cuff was inflated with air, and the cuff pressure was measured by a cuff pressure gauge in both groups (VBM Medizintechnik GmbH, Sulz am Neckar, Germany). Effective ventilation after the airway device insertion was defined as meeting the following criteria: (1) typical square wave capnography, (2) symmetric thoracic expansion (3) absence of audible leak (4) peak inspiratory pressure < 30 mmHg^9^. If the aforementioned criteria were not met, the position of LMA was modified up to 3 times. If the effective ventilation was not achieved, LMA was removed and ETT was intubated with the exclusion of participant from the study. Arterial line was placed in the right radial artery and used it to monitor mean arterial pressure and perform arterial blood gas analysis every 2 h. During surgery, fraction of inspired oxygen (FiO_2_) 0.5, a tidal volume of 8 mL/ideal body weight, and positive end expiratory pressure (PEEP) 6 cmH_2_O were applied in both groups. The respiratory rate was adjusted to maintain the end-tidal carbon dioxide (ETCO_2_) as 35–40 mmHg. After confirming the effective ventilation, a gastric drainage tube was inserted through the gastric access hole of the LMA. When ineffective ventilation was detected during surgery, it planned to remove LMA and replace by ETT. Anesthesia was maintained with isoflurane and continuous remifentanil infusion (up to 0.2 mcg/kg/min) and the intra-abdominal pressure was adjusted at 12 mmHg during laparoscopic procedure. At the end of the surgery, muscle relaxation was reversed with neostigmine 1.5 mg and glycopyrrolate 0.4 mg. The amount of gastric drainage were measured in group LMA. After the airway device was removed, the patient was transferred to the post-anesthetic care unit (PACU).

During anesthetic emergence, the restlessness score (4 = patients are combative, 3 = patients are willing to sit up on the bed without stimulation, 2 = patients move their limbs vigorously without stimulation, 1 = patients are restless without stimulation, and 0 = patients are calm and do not move their limbs without stimulation) was recorded and desaturation (i.e., SpO_2_ less than 92%) was investigated. In the general ward, the degree of sore throat and hoarseness using 4-grade scales (sore throat: 3 = severe, 2 = moderate, 1 = mild, and 0 = no sore throat; hoarseness: 3 = aphonia, 2 = severe, 1 = mild, and 0 = no hoarseness, respectively) were assessed at 6 and 24 h following surgery. Both postoperative nausea and vomiting (PONV) during postoperative day (POD) 1 were recorded as yes or no. Pulmonary aspiration was identified by the radiographic finding with related signs and symptoms including vomiting and dyspnea within POD 3. The grade was recorded as follows: 0 = no pulmonary aspiration, 1 = pulmonary aspiration.

### Outcomes

The primary outcome was the difference in arterial carbon dioxide partial pressure 2 h after the insertion of airway devices (P_a_CO_2__2h). Minute ventilation 2 h after the insertion of airway device (MV_2h) was additionally assessed to minimize the possibility of bias related to the maintenance of predetermined level of ETCO_2_ as 35–40 mmHg. The secondary outcomes were the comparison of hemodynamic parameters (i.e. mean blood pressure [MBP], heart rate [HR], and corrected QT interval [QTc]) and respiratory parameters (i.e. P_a_CO_2_, ETCO_2_, peak inspiratory pressure [PIP], and plateau airway pressure [Ppl]) over time, and postoperative outcomes (sore throat, hoarseness, and PONV), and the incidence of pulmonary aspiration between two groups.

### Statistical analysis

The sample size was calculated to detect equivalence between the use of second-generation LMA and ETT. Based on our pilot data (n = 257, in process*), the patients using second-generation LMA showed the average of PaCO_2_ 39.25 (standard deviation [SD] 3.89) mmHg and the ETT group showed the average PaCO2 39.64 (SD 3.60) mmHg. Two-sided design was used to test equivalence between groups. To analyze the equivalence (margin 3) under power 80%, 82 subjects, 41 subjects per group, were required. Considering a 10% potential failure to complete the protocol, 92 subjects were included.

Schuirmann’s two one-sided tests approach was used to test equivalence of P_a_CO_2__2h and ETCO_2_ between two study groups. Generalized Estimating Equation was applied to repeated measurements of parameters including P_a_CO_2,_ ETCO_2,_ PIP, Ppl, MBP, HR, and QTc. Ordinal data such as postoperative sore throat and hoarseness were compared using Cochran-Armitage trend test. Continuous variables were compared using Student’s *t*-test or the Mann–Whitney U test, as appropriate. Shapiro–Wilk test was used to test normality. Categorical variables were analyzed using Chi-squared test or Fisher’s exact test as appropriate. Data are presented as mean (90% confidence limits [CL] or SD) or median (interquartile range [IQR]) as appropriate for continuous variables; and as number (with percentages) for categorical variables.

All statistical analyses were performed using SAS version 9.4 (SAS Institute Inc, Cary, NC, USA). Two-sided *P-*values were computed and an effect was considered statistically significant at the level of *P* = 0.05.

### Conference presentation

16th congress of the Asian Society of Transplantation 2019, 29th, September, 2019.

## Results

**Figure 1:**
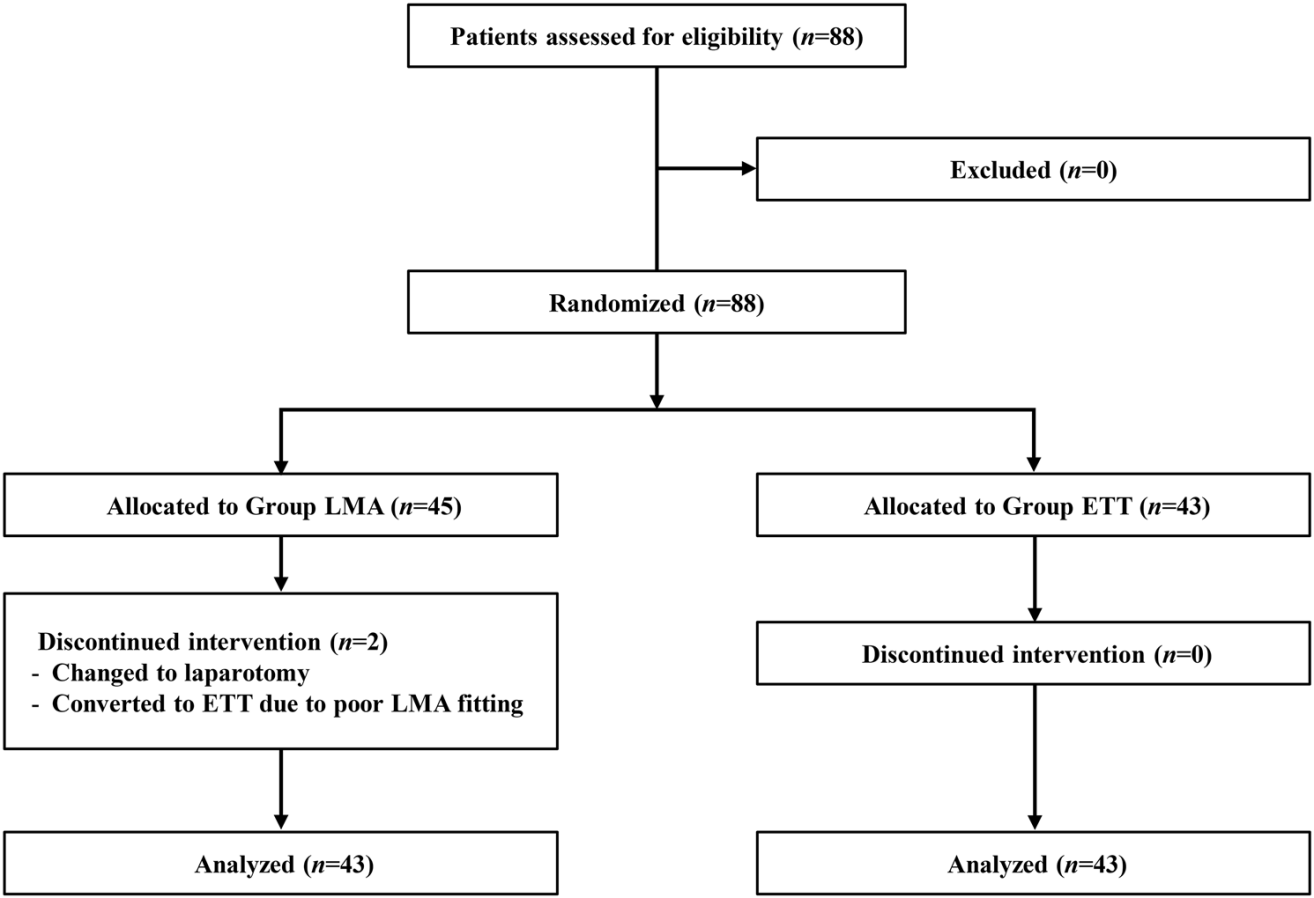
CONSORT diagram. *LMA* laryngeal mask airway, *ETT* endotracheal tube.

Eighty-eight participants were recruited and two cases were subsequently excluded in this trial: one who had switched to insert ETT due to poor LMA fitting despite repeated attempts and one who had converted to laparotomy. Since there were fewer dropouts, a total of 86 participants completed present trial and finished the study earlier than expected (Fig. 1). Participant except for the number of smokers. The proportion of smokers in LMA group was higher than ETT group despite randomization (Table 1).

**Table 1:** Patients and perioperative characteristics.

	Group LMA (*n* = 43)	Group ETT (*n* = 43)
Age (year)	34.1 (11.3)	34.7 (11.7)
Gender (female)	14 (32.6%)	21 (48.8%)
Weight (kg)	64.2 (23.9)	66.2 (9.5)
Height (cm)	166.3 (17.8)	167.1 (7.8)
BMI (kg/m^2^)	22.9 (2.8)	23.7 (2.8)
ASA PS class (I)	36 (83.7%)	37 (86%)
Preoperative hemoglobin (g/dL)	14.5 (1.5)	14.4 (1.4)
**Comorbidity**		
Smoking	11 (25.6%)	4 (9.3%)
Hypertension	2 (4.7%)	2 (4.7%)
Diabetes mellitus	1 (2.3%)	0 (0)

Data are expressed as mean (SD) or number (%).

*LMA* laryngeal mask airway, *ETT* endotracheal tube, *BMI* body mass index, *ASA PS* American Society of Anesthesiologists Physical status.

**Figure 2:**
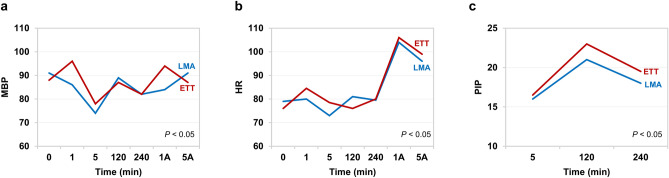
The change in variables over time in both groups. (**a**) The change in mean arterial blood pressure (MBP) over time. (**b**) The change in heart rate (HR) over time. (**c**) The change in peak inspiratory pressure (PIP) over time. *LMA* group LMA, *ETT* group ETT, *1A* 1 min after airway device removal, *5A* 5 min after airway device removal.

The P_a_CO_2__2h showed that LMA and ETT were equally effective (mean difference: 0.99 mmHg, *P* = 0.003; 90% CL − 0.22, 2.19). There was no statistical difference in MV_2h between two groups (group LMA: mean (SD) 5.23 (1.20) L/min vs group ETT: mean (SD) 4.97 (0.84) L/min, *P* = 0.253). The 90% CL of mean difference in ETCO_2__2h was within equivalence margin (mean difference: 0.47 mmHg, *P* < 0.001; 90% CL − 0.22, 1.15). The change in MBP, HR, and PIP were different over time between two groups (*P* < 0.001, *P* = 0.015, and *P* = 0.039, respectively). In particular, group ETT showed significantly elevated intraoperative PIP than group LMA. Aforementioned variables showed smaller changes in group LMA compared to group ETT (Fig. 2). However, the trend of QTc, P_a_CO_2_, ETCO_2,_ and Ppl over time were not different between study groups (*P* = 0.739, *P* = 0.159, *P* = 0.997, and *P* = 0.361, respectively).

Intraoperative and emergence variables were not statistically different between both groups (Table 2). In addition, postoperative upper airway related symptoms such as sore throat and hoarseness, and PONV were comparable between two groups (Table 3). There was no case of pulmonary aspiration in both groups. In group LMA, OLP was 27.10 (8.78) cmH_2_O and the amount of drained gastric contents were 10.10 (15.73) mL. No adverse events were observed in both groups during study period.

**Table 2:** Intraoperative and emergence variables.

	Group LMA (*n* = 43)	Group ETT (n = 43)	*P* value
**Intraoperative data**			
No of airway device insetion attempt	1 (0)	1.02 (0.15)	0.32
Hypoxemia (SpO2 < 92%)	1 (2.3%)	1 (2.3%)	> 0.99
Ineffective ventilation	1 (2.3%)	1 (2.3%)	> 0.99
Anesthesia duration (min)	307.41 (44.76)	302.63 (31.12)	0.566
Airway device maintenance duration (min)	282.4 (31.29)	294.19 (42.94)	0.149
Dissatisfied cases	0 (0)	2 (4.7%)	0.494
**Anesthesia emergence**			
SBP > 160 mmHg	2 (4.7%)	4 (9.3%)	0.676
Restlessness score	0.72 (0.96)	1.02 (1.01)	0.159
PACU staying duration	63.93 (13.63)	66.33 (21.84)	0.543

Data are expressed as mean (SD) or number (%).

*SBP* systolic blood pressure.

**Table 3:** Postoperative variables.

	Group LMA (*n* = 43)	Group ETT (*n* = 43)	*P* value
Sore throat*			
6 h			0.451^^^
Mild	23 (53.49%)	24 (55.81%)	
Moderate	5 (11.63%)	7 (16.29%)	
Severe	1 (2.33%)	1 (2.33%)	
24 h			0.4151^^^
Mild	12 (27.91%)	16 (37.21%)	
Moderate	1 (2.33)	1 (2.33%)	
Severe	0 (0)	0 (0)	
Hoarseness*			
6 h			0.7494^^^
Mild	10 (23.26%)	10 (23.26%)	
Moderate	4 (9.3%)	5 (11.63%)	
Severe	0 (0)	0 (0)	
24 h			0.2143^^^
Mild	3 (6.98%)	9 (20.93%)	
Moderate	2 (4.65%)	2 (4.65%)	
Severe	0 (0)	0 (0)	
**Nausea**			
6 h	28 (65.12%)	27 (62.79%)	0.822^^^^
24 h	14 (32.56%)	18 (41.86%)	0.3722^^^^
**Vomiting**			
6 h	10 (23.26%)	13 (30.23%)	0.465^^^^
24 h	4 (9.3%)	3 (6.98%)	> 0.999^^^^^

Data are expressed as number (%).

*Postoperative sore throat and hoaresness were presented as grading of discomfort.

^^^*P* value, analysis of Cochran-Armitage trend test.

^^^^*P* value, analysis of Chi-squred test.

^^^^^*P* value, analysis of Fisher’s exact test.

## Discussion

Our results demonstrated that second-generation LMA was equivalent to ETT in donors underwent LLDH on intraoperative respiratory, hemodynamic variables, and early postoperative outcomes.

To our knowledge, this is the first randomized controlled trial demonstrated the suitability of second-generation LMA during long-lasting laparoscopic surgery. Although one experimental research investigated the degree of upper airway injury after long-term use (three to 24 h) of second-generation LMA in pigs^10^, most previous human studies reported the mean time of airway device application was about 70–80 min and it did not exceed 100 min^11–13^. Airway devices were maintained approximately 280–290 min in this study. Moreover, unlike previous clinical trials investigated postoperative airway complications including sore throat, dysphagia, and dysphonia after using second-generation LMA and ETT, we compared intraoperative variables in prolonged laparoscopic surgery^11,12,14^. This is another strength of our research.

The most common concerns related to using LMA during laparoscopic surgery are inadequate ventilation, gastric distension, and pulmonary aspiration of gastric contents. Since gastric access port of the second-generation LMA can manage problems related to the gastric distension, improper ventilation may be more important issue in long lasting surgery. Therefore, we focused especially in intraoperative respiratory parameters. As a result, P_a_CO_2,_ which was selected as a marker of the suitability of ventilation, showed that second-generation LMA has an equivalent effect to ETT during LLDH. In addition, our results indicated that ETCO_2_ and Ppl were stable with second-generation LMA. These indicated that both devices might be efficiently used in long-term laparoscopic surgery.

Endotracheal intubation using laryngoscopy is a stressful condition that results in tachycardia and hypertension caused by catecholamine release^12,15–18^. We found that MBP and HR were elevated especially from baseline to 1 min after airway device insertion in the current study. It demonstrated that ETT insertion is more irritating than LMA insertion and cause hemodynamic instability. This result is consistent with previous researches demonstrated that the insertion and removal of the LMA was less invasive and induces fewer stress responses than with ETT^18–20^. Thus, we suggested that second-generation LMA will be more suitable for patients requiring hemodynamic stability.

In the current study, group ETT showed more increased PIP than group LMA during surgery. This is in line with previous studies comparing the airway pressure of LMA and ETT^21,22^. We considered that narrower inner diameter of ETT than that of LMA was related to the increased airway resistance.

We acknowledge several limitations in this study. First, we conducted the trial on liver donors, not patients with certain diseases. Because liver donors are considered to be providing the comparable conditions as healthy volunteers, our results may be different from the diseased patients and may not be applicable to other surgeries. Therefore, we suggest that care should be taken when using LMA for long lasting laparoscopic surgery in patients with various diseases. Second, although there were no cases of pulmonary aspiration in this study, we could not confirm the effectiveness of second-generation LMA on pulmonary complications. Since the number of subjects required in our study was based on PaCO_2_, the primary outcome, it is insufficient to determine difference of pulmonary aspiration between two groups. Further research is needed on the risk of pulmonary aspiration associated with the use of second-generation LMA. Third, the investigator and clinicians in the operating room were not blinded to the assigned group. Since our protocol did not fit for double-blinded, it may cause observer bias. To minimize possible biases, the investigator and clinicians in the operating room decided not to be involved in this trial any more than to fill out information in the operating room. Fourth, since gastric drainage tube was inserted only in group LMA, we did not evaluate the degree of gastric distension between two study groups. There may be a difference because gastric drainage tube was not inserted in group ETT. For accurate comparisons, gastric drainage tube should be inserted in both groups.

In conclusion, second-generation LMA provides appropriate ventilation and stable hemodynamics during LLDH. Second-generation LMA with gastric drainage tube can be selected as one of the effective alternative to ETT for securing airway in patients undergoing prolonged laparoscopic surgery.

## Data Availability

The data that support the findings of this study are available from the corresponding author upon reasonable request.

## Abbreviations

LMA: Laryngeal mask airway
ETT: Endotracheal tube
LLDH: Laparoscopic living liver donor hepatectomy
